# Iron Overload in Chronic Kidney Disease: Less Ferritin, More T2^*^MRI

**DOI:** 10.3389/fmed.2022.865669

**Published:** 2022-03-21

**Authors:** Abdulqadir J. Nashwan, Mohamed A. Yassin, Mohamed Izham Mohamed Ibrahim, Hanan F. Abdul Rahim, Mujahed Shraim

**Affiliations:** ^1^Department of Nursing, Hazm Mebaireek General Hospital, Hamad Medical Corporation, Doha, Qatar; ^2^Hematology and Oncology, Hamad General Hospital, Hamad Medical Corporation, Doha, Qatar; ^3^College of Pharmacy, QU Health, Qatar University, Doha, Qatar; ^4^Department of Public Health, College of Health Sciences, QU Health, Qatar University, Doha, Qatar

**Keywords:** iron overload, T2^*^MRI, serum ferritin, chronic kidney disease, liver iron concentration (LIC)

## Abstract

To date, there is no consensus on the most reliable marker of iron status in patients with chronic kidney disease (CKD). Serum ferritin is used routinely, although it may be a misleading marker for iron overload. The success of T2^*^ MRI in monitoring iron overload in patients with hemoglobinopathies can be beneficial to monitoring patients with CKD.

## Introduction

As anemia represents the feature complication in patients suffering from chronic kidney disease (CKD), most researchers have focused on monitoring anemia rather than iron overload due to iatrogenic iron replacement. Clinicians use serum ferritin routinely to monitor iron status, but serum ferritin is influenced by various factors, including liver disease and inflammation (1). Patients with CKD might suffer sequelae of iron overload affecting the major iron storage sites (2). In the liver, iron overload leads to substantial organ damage and, eventually, liver failure (3). Cardiac iron overload is linked to dysrhythmia and heart failure (4), and iron can build up in the endocrine organs (e.g., thyroid, pancreas, pituitary gland), causing dysfunction as well as delayed puberty in children (5). Other *in vivo* animal models and a few clinical studies also suggest a role for iron overload in brain damage (6, 7). Furthermore, it is known that iron overload makes some microorganisms more virulent. There is mounting evidence suggesting that iron overload promotes the human immunodeficiency virus (HIV) infection progression and may exacerbate the severity of viral hepatitis as well as how well it responds to therapy (8). Importantly, liver and cardiac iron quantification and their correlation with serum ferritin levels is not well-researched in patients with CKD compared to patients with hemoglobinopathies. As a result, there is no clear cutoff value for ferritin in the existing clinical guidelines, necessitating the identification of more reliable iron overload diagnostics.

## When Less is More

The global prevalence of CKD ranges between 11.7 and 15.1%, which represents a global public health issue (9). CKD claimed 1.2 million lives in 2017 and was the world's 12th largest cause of mortality. In addition, CKD was responsible for 35.8 million disability-adjusted life years (DALYs), whereas CVD was responsible for 25.3 million DALYs in the same year. Diabetes is the major cause of CKD DALYs, accounting for 30.7 percent of all cases (10). Furthermore, CKD directly impacts the morbidity and mortality rates through its progression to cardiovascular disorders and end-stage renal disease (ESRD) (11).

Anemia (*defined as hemoglobin [Hb]*<*11 g/dL in women and*<*12 g/dL in men*) is a known consequence of CKD with a prevalence of <10% in stages I & II, about 20–40% in stage III, and 50–60% in stage IV. However, due to the estimated glomerular filtration rate (eGFR) decline, the prevalence exceeds 70% in stage V (12, 13).

## Consequences of Iron Overload

Iron overload may affect the liver (e.g., cirrhosis which increases the risk of hepatocellular carcinoma) and the heart (e.g., heart failure and arrhythmias), and it may lead to endocrine and metabolic complications, such as hypogonadism and diabetes mellitus, in addition to other musculoskeletal and skin-related complications (14). Furthermore, higher iron reserves in the body may shift the immunoregulatory balance negatively, compromising the immune system and complicating therapeutic management of underlying acute and chronic illnesses (15). According to recent case reports, iatrogenic iron replacement may lead to hemochromatosis (secondary iron overload) in patients with CKD, which represents an “emerging medical challenge” (16, 17). Unfortunately, patients with CKD have few alternatives for treating hemochromatosis due to the poor creatinine clearance for most iron chelators (18). This poses a clinical quandary when balancing the necessity to correct iron deficiency anemia while preventing iron overload (19).

## Iron Markers in CKD

### “Old Is Gold”

The Liver, bone marrow (BM), and spleen ate the primary iron storage sites. Consequently, the liver is the first organ to exhibit signs of excess iron. Thus, liver or bone marrow biopsy has long been considered the gold standard for identifying and measuring iron levels, but with disadvantages, including invasiveness, inconvenience, and impracticality (20).

### “Beautiful Deception”

According to CKD's latest clinical guidelines, “The Kidney Disease: Improving Global Outcomes (KDIGO) 2012,” transferrin saturation (TSAT) and serum ferritin levels are recommended for tailoring iron therapy (1, 16, 19, 21, 22). However, the accuracy of TSAT and serum ferritin and even serum Fe as hepatic or cardiac iron overload indicators is questionable, since they are influenced by several factors, including malnutrition, liver disease, and inflammation (23). For instance, the association between serum ferritin and liver iron concentration (LIC) is well studied in hemoglobinopathies [e.g., transfusion-dependent (TD) beta-thalassemia major (BTM) and sickle cell disease (SCD)] (24). However, this association may not be generalized to patients with CKD without robust scientific evidence due to the nature of the disease and the mechanism of body iron metabolism and overload.

### “Leave No Stone Unturned”

Other iron markers are either still under investigation, or limited to certain institutions because of cost and clinical feasibility, or have unknown cutoff levels, such as superconducting quantum interference device (SQUID), percentage of hypochromic red cells (PHRC), the content of hemoglobin reticulocyte (CHr), erythrocyte zinc protoporphyrin (ZnPP), and Soluble transferrin receptor (sTfR). For example, in 2004, a report was conducted that included SQUID assessments of hepatic iron levels in 40 patients on hemodialysis receiving IV iron. Although 30% of the patients had serum ferritin levels >500 ng/mL, this investigation found that the other 70% had indications of mild to severe iron overload (25). Although SQUID is typically reliable and reproducible, there are presently a limited number of operational devices worldwide, making it unsuitable for clinical use (26).

## Is T2^*^MRI a Remedy?

Magnetic resonance imaging (MRI) such as T2^*^ is commonly used for hepatic, cardiac, and pancreatic iron overload monitoring and demonstrates a good correlation with liver biopsy results in hemoglobinopathies, including monitoring of treatment response to intravenous iron replacement (27, 28). T2^*^MRI is a preferred modality, particularly in individuals who have contraindications to liver biopsy or when a quantitative measurement of liver iron content cannot be conducted. In patients with BTM, MRI is usually indicated based on several factors (29), such as the frequency of blood transfusions (>20 times), the accumulative dose of iron supplements (if prescribed 5 times per year), or the serum ferritin levels (if exceeding 1,000 ng/mL). MRI in patients with BTM is recommended on an annual basis and twice per year in patients with severe or very severe liver iron overload (30). However, in patients with CKD, there are no published guidelines or recommendations on the criteria for conducting T2^*^MRI, including frequency.

Several studies indicate that T2^*^MRI is the “best” non-invasive approach for diagnosing liver iron overload, determining severity, and monitoring response to therapy (serial evaluation) with a high accuracy level (31–34). In 2012, significant iron overload (serum ferritin >1,000 ng/mL) in the liver and spleen was recently observed in more than 90% (*N* = 21) of hemodialysis patients on IV iron therapy (22). On the other hand, Rostoker et al. (35) investigated 119 hemodialysis patients receiving erythropoiesis-stimulating agents (ESA) and iron therapy and assessed their LIC using T2^*^ MRI. Mild to severe hepatic iron excess was seen in 84 percent of the patients, with 36 percent having severe iron overload similar to that seen in hereditary hemochromatosis. Therefore, the iron concentration of the liver (LIC) is highly linked to the total iron dosage taken. According to a landmark paper by Locatelli et al. (36) “Despite the fact that excess iron in the liver is potentially harmful, the clinical consequences of high iron content estimated by magnetic resonance is not known.” The paper also pointed to the lack of clear evidence indicating an upper limit at which ferritin level that can be considered safe. In addition to the clinical validity and feasibility, the availability and access to MRI technology and time and cost factors are challenging in many underprivileged settings.

## Outstanding Questions

Thus, to know the unknown, detecting and quantifying hepatic and cardiac iron excess is vital for initiating iron therapy and preventing iron excess in patients with CKD. Furthermore, establishing a safe cutoff level of serum ferritin by correlating with T2^*^MRI in patients with CKD is an emerging unmet need.

## Author Contributions

AN: conceptualization. AN, MY, MM, HA, and MS: literature search and manuscript preparation (draft and final editing). All authors read and approved the final manuscript.

## Conflict of Interest

The authors declare that the research was conducted in the absence of any commercial or financial relationships that could be construed as a potential conflict ofinterest.

## Publisher's Note

All claims expressed in this article are solely those of the authors and do not necessarily represent those of their affiliated organizations, or those of the publisher, the editors and the reviewers. Any product that may be evaluated in this article, or claim that may be made by its manufacturer, is not guaranteed or endorsed by the publisher.
